# Therapist Feedback and Implications on Adoption of an Artificial Intelligence–Based Co-Facilitator for Online Cancer Support Groups: Mixed Methods Single-Arm Usability Study

**DOI:** 10.2196/40113

**Published:** 2023-06-09

**Authors:** Yvonne W Leung, Steve Ng, Lauren Duan, Claire Lam, Kenith Chan, Mathew Gancarz, Heather Rennie, Lianne Trachtenberg, Kai P Chan, Achini Adikari, Lin Fang, David Gratzer, Graeme Hirst, Jiahui Wong, Mary Jane Esplen

**Affiliations:** 1 de Souza Institute University Health Network Toronto, ON Canada; 2 Department of Psychiatry Temerty Faculty of Medicine University of Toronto Toronto, ON Canada; 3 College of Professional Studies Northeastern University Toronto, ON Canada; 4 Department of Psychology University of Toronto Toronto, ON Canada; 5 BC Cancer Agency Vancouver, BC Canada; 6 Centre for Psychology and Emotional Health Toronto, ON Canada; 7 Centre for Data Analytics and Cognition La Trobe University Melbourne Australia; 8 Factor-Inwentash Faculty of Social Work University of Toronto Toronto, ON Canada; 9 Centre for Addiction and Mental Health Toronto, ON Canada; 10 Department of Computer Science University of Toronto Toronto, ON Canada

**Keywords:** cancer, recommender system, natural language processing, LIWC, natural language processing, emotion analysis, therapist adoption, therapist attitudes, legal implications of AI, therapist liability

## Abstract

**Background:**

The recent onset of the COVID-19 pandemic and the social distancing requirement have created an increased demand for virtual support programs. Advances in artificial intelligence (AI) may offer novel solutions to management challenges such as the lack of emotional connections within virtual group interventions. Using typed text from online support groups, AI can help identify the potential risk of mental health concerns, alert group facilitator(s), and automatically recommend tailored resources while monitoring patient outcomes.

**Objective:**

The aim of this mixed methods, single-arm study was to evaluate the feasibility, acceptability, validity, and reliability of an AI-based co-facilitator (AICF) among CancerChatCanada therapists and participants to monitor online support group participants’ distress through a real-time analysis of texts posted during the support group sessions. Specifically, AICF (1) generated participant profiles with discussion topic summaries and emotion trajectories for each session, (2) identified participant(s) at risk for increased emotional distress and alerted the therapist for follow-up, and (3) automatically suggested tailored recommendations based on participant needs. Online support group participants consisted of patients with various types of cancer, and the therapists were clinically trained social workers.

**Methods:**

Our study reports on the mixed methods evaluation of AICF, including therapists’ opinions as well as quantitative measures. AICF’s ability to detect distress was evaluated by the patient's real-time emoji check-in, the Linguistic Inquiry and Word Count software, and the Impact of Event Scale-Revised.

**Results:**

Although quantitative results showed only some validity of AICF’s ability in detecting distress, the qualitative results showed that AICF was able to detect real-time issues that are amenable to treatment, thus allowing therapists to be more proactive in supporting every group member on an individual basis. However, therapists are concerned about the ethical liability of AICF’s distress detection function.

**Conclusions:**

Future works will look into wearable sensors and facial cues by using videoconferencing to overcome the barriers associated with text-based online support groups.

**International Registered Report Identifier (IRRID):**

RR2-10.2196/21453

## Introduction

Half of all Canadians will be diagnosed with cancer in their lifetime, and the illness is often associated with psychological distress. Canadians living in remote areas have limited access to supportive services, and many experience difficulties in accessing services due to physical disabilities. The recent onset of the COVID-19 pandemic and the social distancing requirement have created a further demand for virtual support programs [1].

Emerging evidence supports the effectiveness of online support groups to reduce access barriers [2]. CancerChatCanada offers therapist-led, text-based online support groups to address patients’ cancer-related distress and has demonstrated positive results. CancerChatCanada, offered by de Souza Institute, consists of a series of synchronized, therapist-led, text-based online support groups for patients with cancer and their caregivers. CancerChatCanada is a national program operated in collaboration with 6 provincial cancer agencies in Canada. The online support groups vary in theme and therapeutic model, with all groups being manual-based and consisting of 8-10 sessions [3]. During the group sessions, the facilitators aim to support and process discussions based on session themes and related concerns while also acknowledging and attending to the members’ emotional needs individually. Each online support group is led by 1 or 2 licensed counselors/therapists and is composed of 6-10 participants, meeting weekly for 8 weeks in a web-based synchronous chatroom. However, therapists leading text-based online support groups often find it challenging to address individual group members’ simultaneous responses around their distress/needs in the absence of visual communicative cues. Recent advances in artificial intelligence (AI) may offer novel solutions. Using typed texts from online support groups, AI can monitor therapy sessions, help identify the potential risk of mental health concerns, alert group facilitator(s), and automatically recommend tailored resources while monitoring group emotions. In particular, 1 study has developed an AI system to analyze therapy session transcripts to provide a cognitive behavioral therapy session fidelity score for therapists [4].

**Figure 1:**
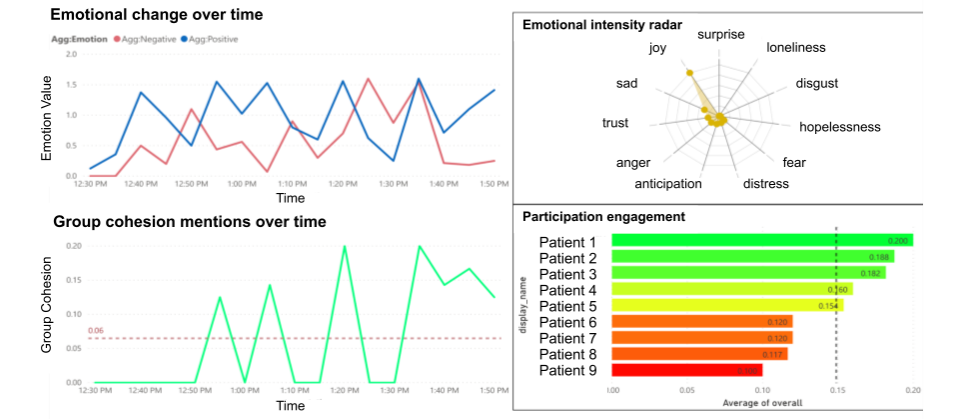
Dashboard of the group and individual emotion analysis. Agg: aggregate.

**Figure 2:**
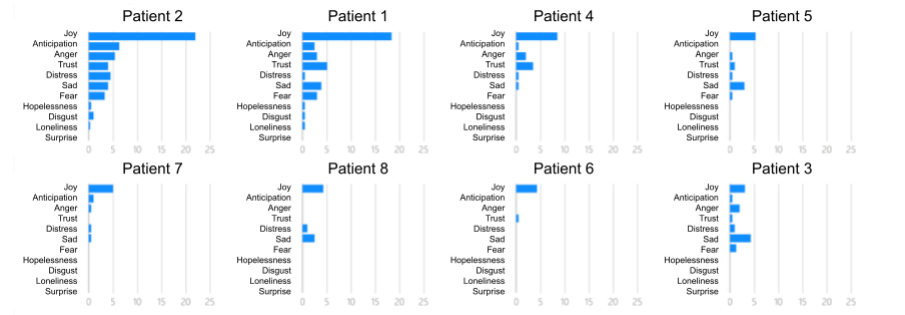
Breakdown of the emotion analysis of individuals.

**Figure 3:**
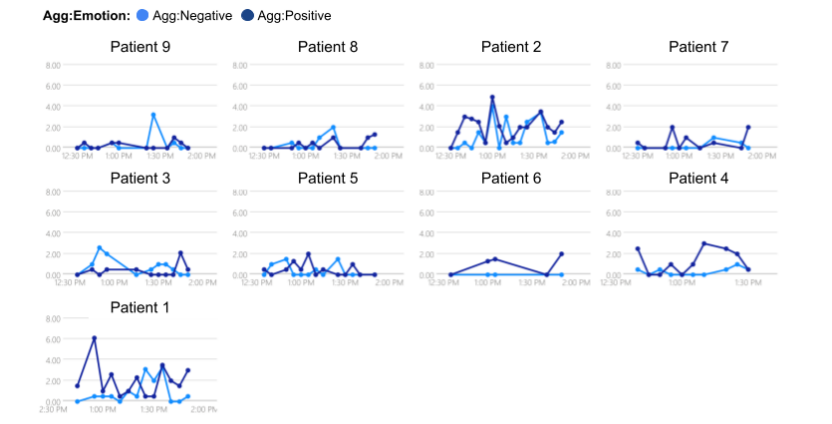
Positive and negative emotion analyses of individuals. Agg: aggregate.

**Figure 4:**
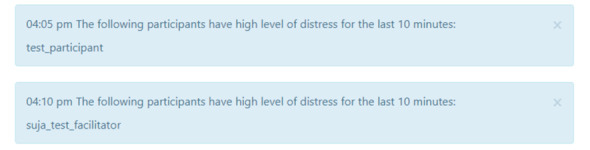
Distress warnings for therapists.

**Figure 5:**
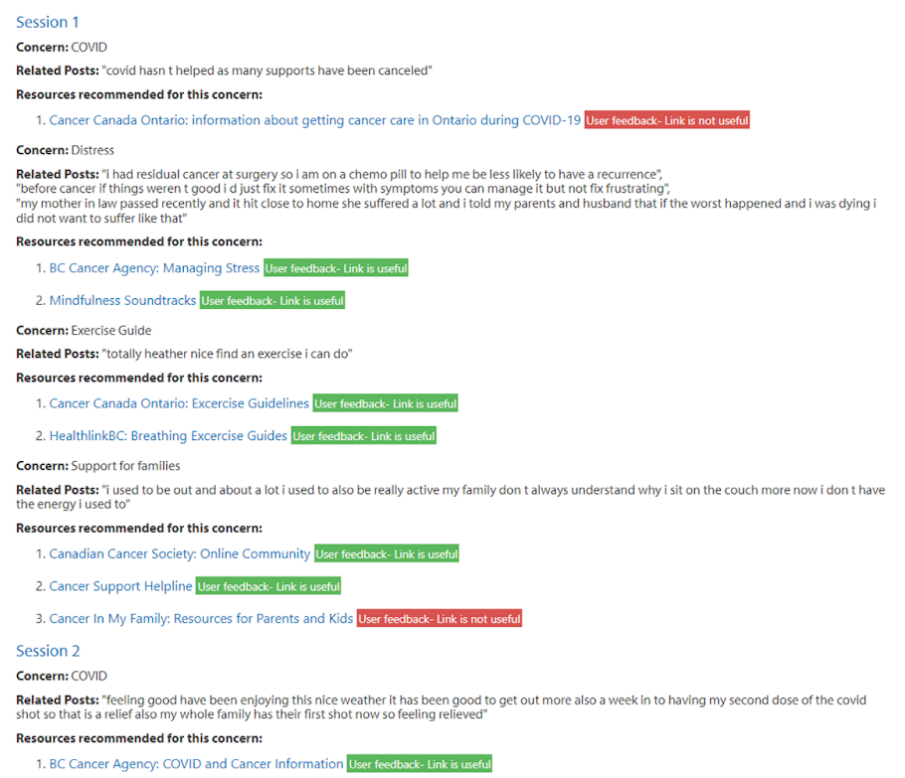
Participant conversation summary and resource recommender.

We developed and evaluated an AI-based co-facilitator (AICF) to track and monitor online support group participants’ distress through a real-time analysis of texts posted during online support group sessions. Specifically, AICF was designed for the following functions: (1) profiling, that is, generate participant profiles with discussion topic summaries and emotion trajectories for each session in a dashboard (Figures 1-3), (2) distress warning, that is, identify participant(s) at risk for increased emotional distress and alert the therapist for follow-up (Figure 4), and (3) resource recommendation, that is, automatically suggest tailored resources based on participant needs (Figure 5). AICF allows real-time detection of issues (eg, disengagement, feeling unsupported) that were amenable to treatment, allowing therapists to be more proactive in supporting group members on an individual basis during the group sessions. A full protocol of the AICF algorithm development and preliminary findings has been published previously [3,5]. The AICF development details are shown in Multimedia Appendix 1 [6-25].

The objectives of this study were to present the results of therapist user testing and their experiences by using focus group methodology. The detailed training and testing results of each AICF functionality will be published in detail in a separate paper.

## Methods

### Study Participants

Eleven therapists and 156 participants in the online support group were recruited through CancerChatCanada as well as through the web page and social media accounts (Facebook and Twitter) of de Souza Institute. Patients with a cancer diagnosis were included in this study. Distressed patients who needed immediate psychological care were excluded. The therapists were mainly social workers, with 1 registered clinical psychologist and 1 registered clinical counsellor. There were no exclusion criteria for the therapists.

### Ethics Approval

This study was approved by the University Health Network research ethics board (18-5354).

### Study Design

This is a mixed methods, single-arm study that evaluated AICF’s feasibility, acceptability, validity, and reliability among CancerChatCanada therapists and participants. The feasibility and acceptability of AICF were assessed by a focus group composed of therapists with a designed interview guide (Multimedia Appendix 2). The validity and reliability of AICF were assessed using correlation statistics. This study was conducted from July 1, 2019, to August 31, 2021.

### Procedure

AICF was deployed and tested in the back end (out-of-the therapists’ view) in 3 online support groups and with beta testing in 10 groups. The AICF system developed in phase I of our research provided outputs that included 8 basic emotions (sadness, anger, fear, joy, trust, surprise, anticipation, disgust) and their intensities, group cohesion, engagement, and emotional profiling features [3]. In our quantitative evaluation, we hypothesized that AICF would have high correlations with standard measures of distress, high performance of distress threshold (area under the curve>70%), and predictive values for distress. Upon completing user testing, 3 therapists were interviewed in a focus group, which involved 4 parts of a discussion: (1) review the study purposes and design specifications, (2) distress and other emotions, (3) recommendations for specific functionalities, and (4) overall impression of AICF.

### Measures

A standardized measure called the Impact of Event Scale-Revised (IES-R) was used to validate AICF’s ability in detecting the distress of online support group participants. The IES-R [26] was used to measure cancer-related distress and deployed before and after the online support group program and is a 22-item measure rated on a 5-point Likert scale, yielding a total score ranging from 0 to 88. The IES-R has 8 items on the subscale for intrusion (Cronbach α=.87-.94), 8 items for avoidance (Cronbach α=.84-.87), and 6 items on hyperarousal (Cronbach α=.79-.91).

### Analysis

#### Quantitative Analysis

We defined distress as aggregating all the negative emotions (sadness, anger, fear, disgust) detected by AICF. To test the validity of AICF in detecting distress, we designed a real-time emoji check-in to gain insights from the participants directly during group sessions. Nine common emojis (neutral, happy, feeling supported, relaxed, anger, bored, overwhelmed, sad, or worried) would pop up on the participant screen every 30 minutes during the 1.5-hour online support group session. The Linguistic Inquiry and Word Count (LIWC) software [27] was applied to all textual data to obtain a reference score for positive and negative emotions. LIWC scanned each line of the conversation for positive and negative emotions. We hypothesized that correlations between LIWC and AICF outputs would be strong (≥0.7).

To validate AICF by the close to real-time participant emotional states, we grouped the 9 emojis into positive and negative states, with the neutral emoji excluded. The remaining 8 emojis were grouped into positive (happy, feeling supported, relaxed) and negative (anger, bored, overwhelmed, sad, worried) emotions. The number of positive and negative emojis for each participant was counted, and their averages were calculated for each 30-minute interval. For example, if the participant chose happy, feeling supported, worried, or sad, their positive emotion count would be 2/3 and negative count would be 2/5. A Spearman ρ correlation test was conducted on the average positive and negative emoji counts against positive and negative scores produced by LIWC and AICF. The fourth session was selected for the analysis, as the group would have developed a reasonable level of rapport and trust among the members and the facilitator by then. Construct (convergent) validity of AICF-detected distress was compared against the self-reported standardized measure (IES-R). We hypothesized that AICF-extracted negative emotions in the fourth session were positively correlated with the distress level after the program, as assessed by IES-R.

#### Qualitative Analysis

The focus group qualitative data were analyzed by content analysis [28] completed by 2 members of the research team (SN and YL). The questions were designed to ask about the opinion of each functionality of AICF. We extracted key themes from each question discussion and categorized them into pros and cons of each functionality of AICF and created a table to display the extracted themes with therapist quotes. When there were disagreements between the 2 members, a third person (LD) would resolve the conflicts by revising the wordings upon which all parties agreed. The results were ordered from high to low frequency of mentions.

## Results

### Participant Characteristics

Table 1 shows the characteristics of the participants in the CancerChatCanada online support groups; 156 participants consented and completed the pre–group surveys, while 91 participants participated in the fourth session and 77 participated in the last session. Five groups (active treatment, COVID anxiety, advanced cancer, active treatment, active cancer support) were removed, as the AICF algorithm was being tested and updated (n=57).

The F1-scores for distress detection, group cohesion, and resource recommendation were 0.71, 0.80, and 0.88, respectively. AICF-detected distress showed consistent but weak correlations with patient-selected negative emojis in the first 30 minutes (Table 2) and during 30-60 minutes of the session (*r*=0.29, *P=*.004; *r*=0.21, *P=*.004, respectively). There were moderate correlations between AICF distress and LIWC negative emotions (*r*=0.39, *P<*.001; *r*=0.51, *P<*.001) in the second (Table 3) and last 30 minutes of the session (Table 4). There were no relationships between AICF distress in the fourth session and the standardized measure of distress (IES-R) in the pre–group survey (*P=*.35).

**Table 1:** Characteristics of the participants (N=156).

Characteristic		Values, n (%)
**Age group (years)**		
	25-34	9 (5.8)
	35-44	21 (13.5)
	45-54	39 (25)
	55-64	59 (37.8)
	≥65	28 (18)
**Province**		
	Alberta	17 (10.9)
	British Columbia	59 (37.8)
	Ontario	62 (39.7)
	Other	18 (11.5)
**Cancer type**		
	Breast	76 (48.7)
	Colorectal	11 (7.1)
	Gastrointestinal	5 (3.2)
	Gynecological	8 (5.1)
	Non-Hodgkin lymphoma	10 (6.4)
	Other	46 (29.5)
**Support group**		
	Active treatment	35 (22.4)
	Advanced cancer	19 (12.2)
	Caregivers	23 (14.7)
	Expressive arts	7 (4.5)
	Fear of cancer recurrence	18 (11.5)
	Posttreatment	15 (9.6)
	Restoring body image and sexual health after cancer	24 (15.4)
	COVID-related anxiety	15 (9.6)

**Table 2:** The Spearman ρ correlations among artificial intelligence–based co-facilitator, Linguistic Inquiry and Word Count, and emoji scale during the first 30 minutes of session 4.

Variable		Positive emotion (Human)	Negative emotion (Human)	Positive emotion (AICF^a^)	Negative emotion (AICF)	Positive emotion (LIWC^b^)	Negative emotion (LIWC)
**Positive emotion (Human)**							
	*r*	1	–0.449	0.264	–0.154	–0.112	–0.052
	*P* value	—^c^	<.001	.009	.13	.27	.61
**Negative emotion (Human)**							
	*r*	–0.449	1	0.043	0.293	0.015	0.141
	*P* value	<.001	—	.67	.004	.88	.17
**Positive emotion (AICF)**							
	*r*	0.264	0.043	1	0.299	0.063	0.037
	*P* value	.009	.67	—	.003	.54	.72
**Negative emotion (AICF)**							
	*r*	–0.154	0.293	0.299	1	–0.061	0.17
	*P* value	.13	.004	.003	—	.55	.10
**Positive emotion (LIWC)**							
	*r*	–0.112	0.015	0.063	–0.061	1	0.480
	*P* value	.27	.88	.54	.55	—	<.001
**Negative emotion (LIWC)**							
	*r*	–0.052	0.141	0.037	0.17	0.480	1
	*P* value	.61	.17	.72	.10	<.001	—

^a^AICF: artificial intelligence–based co-facilitator.

^b^LIWC: Linguistic Inquiry and Word Count.

^c^Not applicable.

**Table 3:** The Spearman ρ correlations among artificial intelligence–based co-facilitator, Linguistic Inquiry and Word Count, and emoji scale during the second 30 minutes of session 4.

Variable			Positive emotion (Human)		Negative emotion (Human)		Positive emotion (AICF^a^)		Negative emotion (AICF)		Positive emotion (LIWC^b^)		Negative emotion (LIWC)
**Positive emotion (Human)**													
	*r*	1		–0.643		0.075		–0.153		–0.012		–0.191	
	*P* value	—^c^		<.001		.48		.14		.91		.07	
**Negative emotion (Human)**													
	*r*	–0.643		1		–0.077		0.205		–0.057		0.186	
	*P* value	<.001		—		.46		.048		.59		.07	
**Positive emotion (AICF)**													
	*r*	0.075		–0.077		1		0.212		0.193		0.075	
	*P* value	.48		.46		—		.04		.06		.47	
**Negative emotion (AICF)**													
	*r*	–0.153		0.205		0.212		1		0.146		0.390	
	*P* value	.14		.048		.04		—		.16		<.001	
**Positive emotion (LIWC)**													
	*r*	–0.012		–0.057		0.193		0.146		1		0.403	
	*P* value	.91		.59		.06		.16		—		<.001	
**Negative emotion (LIWC)**													
	*r*	–0.191		0.186		0.075		0.390		0.403		1	
	*P* value	.07		.07		.47		<.001		<.001		—	

^a^AICF: artificial intelligence–based co-facilitator.

^b^LIWC: Linguistic Inquiry and Word Count.

^c^Not applicable.

**Table 4:** The Spearman ρ correlations among artificial intelligence–based co-facilitator, Linguistic Inquiry and Word Count, and emoji scale during the last 30 minutes of session 4.

Variable			Positive emotion (Human)		Negative emotion (Human)		Positive emotion (AICF^a^)		Negative emotion (AICF)		Positive emotion (LIWC^b^)		Negative emotion (LIWC)
**Positive emotion (Human)**													
	*r*	1		–0.564		–0.004		–0.079		0.015		–0.182	
	*P* value	—^c^		<.001		.97		.45		.89		.08	
**Negative emotion (Human)**													
	*r*	–0.564		1		0.021		0.167		–0.099		0.122	
	*P* value	<.001		—		.84		.11		.34		.24	
**Positive emotion (AICF)**													
	*r*	–0.004		0.021		1		0.093		0.064		0.067	
	*P* value	.97		.84		—		.37		.54		.52	
**Negative emotion (AICF)**													
	*r*	–0.079		0.167		0.093		1		0.032		0.505	
	*P* value	.45		.11		.37		—		.76		<.001	
**Positive emotion (LIWC)**													
	*r*	0.015		–0.099		0.064		0.032		1		0.481	
	*P* value	.89		.34		.54		.76		—		<.001	
**Negative emotion (LIWC)**													
	*r*	–0.182		0.122		0.067		0.505		0.481		1	
	*P* value	.08		.24		.52		<.001		<.001		—	

^a^AICF: artificial intelligence–based co-facilitator.

^b^LIWC: Linguistic Inquiry and Word Count.

^c^Not applicable.

### Focus Group Participants

Four female therapists from CancerChatCanada participated in our focus group. Each therapist had more than 2 years of facilitating online text-based support groups. In addition, all therapists had a social work background. The therapists conducted online support groups using AICF. Table 5 shows the focus group findings summarized by the functions of AICF and their pros and cons: emoji check-in, engagement score, distress warning, cohesion score, resource recommender, and dashboard. Each of these functions are detailed below.

**Table 5:** Focus group result summary.

AICF^a^ function	Representable pros	Representable cons	Therapist quotes for pros	Therapist quotes for cons
Emoji check-in	Emoji check-in provided facilitators sufficient feedback on participant emotions to address the absence of visual cues. Emoji check-in produced less invasive but critical information than a distress warning. Emoji check-in helped bridge the gap between the postsession report and clinical outcomes such as engagement and distress.	Emoji check-in results were not shown in the session in real time, limiting facilitators’ situational awareness. Facilitators did not have control over the deployment of emoji check-in when needed.	…*The emojis help address the lack of non-verbal feedback.* [Therapist 3] …*The emoji check-in helped provide more granular information regarding distress compared to distress warnings.* [Therapist 3] …*Sometimes the post-session reports don’t line up with the red bar or other analyses, however, emojis help address this gap.* [Therapist 1]	…*Facilitators can’t see the emojis during the session, so participants might feel ignored if their concerns aren’t being taken seriously.* [Therapist 3] …*It would be beneficial if we could deploy the emoji check-in when we believe it is appropriate.* [Therapist 3]
Engagement score	Engagement score was helpful in identifying inactive participants.	The system could not show the typing of participants as an engagement indicator. If a participant left early, they would be flagged as low engagement. Text might be insufficient to show engagement as participants were thinking or feeling beyond what they could express in text. Other indicators such as heart rate might be complementary to the text-based analysis.	…*The engagement score is really helpful to see who is actually inactive.* [Therapist 1]	…*I would love to see the participant typing.* [Therapist 2] …*Is there a way the system knows if the person has left early and is not just disengaged?* [Therapist 1] …*Engagement isn’t always shown through text. What someone is thinking or feeling beyond what text messages they are sending.* [Therapist 1]
Distress warning	Facilitator felt they could provide more support to participants with distress warnings during the session. Distress warnings provided a reminder for the facilitator to follow up with the distressed patient.	Distress warnings could not help therapists to distinguish between healthy and unhealthy positivity, which refers to participants who refused to acknowledge their negative emotions. The distress warning score needed fine-tuning as most participants were usually above average. The distress graph needs to be simplified.	…*I had a patient with a distress warning, so I directed the group to provide more support. I was really happy for the group support.* [Therapist 1] …*If I see the distress warning, it reminds me to follow up with them after the session.* [Therapist 1]	…*When a participant was showing toxic positivity, their messages were still read as “positive.”* [Therapist 1] …*The system needs improvement on setting an average, since most participants were above the red bar.* [Therapist 1] …*Make the distress graph easier to read.* [Therapist 2]
Group cohesion score	The group cohesion score was helpful and is relative to other participants.	There were some discrepancies between the cohesion score and facilitator's judgement or experience concerning group cohesion.	…*It is helpful that the group cohesion scores are relative to other participants.* [Therapist 1]	…*A recent session I facilitated had a red cohesion score, however, this feedback does not fit with my experience with the group.* [Therapist 1]
Resources recommender	The recommender system could standardize the distribution of reference materials to participants to maintain group cohesion and fluidity. The recommender system was helpful to track participants’ progress on the reference materials and their ratings on the usefulness.	Facilitators preferred to read and add additional materials into the automated email content before sending to patients.	…*I want everyone to read the same material, it can help improve group cohesion and fluidity.* [Therapist 3] …*It’s really handy to see if participants have opened and clicked on the material and I can see whether or not it’s useful.* [Therapist 2]	…*Sometimes there are resources I want to add, but I don’t want to send them another email on top of the automated email.* [Therapist 2]
Dashboard	The conversation summary on the dashboard effectively summarized patient emotions and concerns.	The distress graph was visually overwhelming as it showed the status of all participants. It was recommended that only abnormal distress levels be shown during the session. Facilitators suggested the need for a more succinct summary with the most critical information.	…*The conversation summary was useful to look at patients’ feelings and concerns during the session.* [Therapist 1]	…*It’s [distress graph] visually busy. Unless there is someone whose fluctuating out of the usual boundary leaves it out maybe, when in range it’s not too important to know.* [Therapist 2] …*The summary should only present the most important information and put the other details somewhere else.* [Therapist 2]

^a^AICF: artificial intelligence–based co-facilitator.

### Focus Group Findings

#### Emoji Check-in

When participants were asked about their preferences of AICF, the majority of the comments positively addressed the emoji check-in as nonverbal feedback from the group participants. The emoji check-in was in fact a non-AI function implemented to give researchers the reference point for AICF’s real-time emotional tracking. Emojis supplemented the nonverbal clues absent from web-based settings by providing information on each participant's emotions in a simple manner, indicating when participants needed additional support. The therapists generally preferred emoji check-in over distress warnings, as “the distress warning makes you feel that you have missed something.” They regarded distress warnings as possibly increasing pressure and cognitive load on the therapist while facilitating the group session. The emoji check-in function also received many suggestions for further refinement. For example, the dashboard could include emoji check-in results and statistics for instant review. Some therapists expressed that the patient’s emoji status could be shown in real time on the therapist screen to allow for a better understanding of the emotional status of each participant. Others expressed that adding an emoji check-in only at the end of each session could help assess the patient's satisfaction. Lastly, several therapists wished that they had the ability to deploy emoji check-ins whenever they wanted.

#### Engagement Score

Several comments from the focus group positively addressed the engagement score function. One therapist shared that the function helped indicate which patients were inactive, as the absence of visual cues made it difficult to judge participant engagement during sessions where the group members are receiving multiple texts. Therapists also appreciated the non-AI function of the engagement alert that flagged those patients who were inactive for over 10 minutes; therapists could immediately attend to the inactive patient. The engagement score provided after the session also provided an important indicator for facilitators to gauge patient engagement. For improvement, 1 therapist respondent proposed that the group facilitator should be able to chat with participants privately during the group session in order to increase engagement. Another recommended introducing an additional alert to the group facilitator when participants dropped out of the session. Some therapists felt that evaluating the engagement by using textual data could be insufficient, as participants may be thinking or feeling something beyond what they could text. To overcome this issue, it was recommended that patients wear a sensor to monitor biometric signs such as heart rate during the session, which may produce a more accurate engagement score. Lastly, some advised that the chatroom could include read receipts and typing-in-real-time indicators as a measurement of engagement.

#### Distress Warning

Therapists positively evaluated the distress warning function. They shared that the warning drew their attention to distressed patients, and they were able to provide proper support to the patients during the session in a timely fashion. They also appreciated that patient distress recorded in the session summary assisted them in accurately evaluating their group participants for necessary follow-up.

A therapist suggested that the distress graph could be represented in a more succinct manner—flagging only those who displayed extremely high levels of distress that warranted clinical actions. One therapist worried about the legality issues, for example, if the distress warning could be held as evidence against the therapists for potential negligence if something terrible happened to the patient. As distress was common in online support groups, these warnings could add extra pressure to the therapists. Therefore, therapists suggested including a disclaimer to protect them from being accused of malpractice. Similar to other clinical settings, online support groups are a nonemergency service where clinicians are not expected to respond to or to rule out every possible self-harm warning sign. Future studies should explore ways to reduce the ethical liability for therapists when using AI-generated distress warnings.

#### Group Cohesion Score

The therapists described the cohesion score as being helpful to demonstrate how well patients felt being connected with each other in the group. They expressed that a high cohesion score was a true indication that patients were more active and attentive during the session, increasing overall patient satisfaction, better experience, and greater support group effectiveness.

One therapist mentioned that there was some inconsistency between the group cohesion score and her own observations. Another therapist suggested designing an option to filter out absent participants when calculating the group cohesion score. Others also recommended that the facilitator should have the ability to rate group cohesion as a way to validate and calibrate the AICF-generated cohesion score. Another found that the positivity detected by AI was indistinguishable from toxic positivity, which refers to the inability to express negative emotional expressions encouraged by the therapist [29]. Indeed, they commented that toxic positivity could adversely impede group cohesion development, as participants would refrain from expressing their negative emotions if the overall tone was highly positive.

#### Resource Recommender

The therapists uniformly acknowledged that the resource recommender detected issues mentioned by the participants in a timely manner and therefore reduced their workload by providing relevant resources tailored for patients at the end of each session. One therapist suggested that all participants should have access to common materials aligned to a specific theme of each session to enhance group cohesion and fluidity. Several therapists also suggested that the host should be able to add additional web-based resources to the recommender system and edit the AICF-generated email containing the recommendation prior to sending to patients.

#### Dashboard

The therapists overall liked the AICF summary of concerns for each patient provided in the dashboard. However, there were some additional suggestions for improvement of the dashboard. One therapist expressed that the information on the dashboard could be more succinct. Other therapists commented that the dashboard should prioritize information and display more essential items first, for example, the group cohesion score. In addition, another therapist suggested that a graphical display of individual distress data across the sessions and flagging only the extremely distressed individual would facilitate clinical responses.

#### Videoconferencing

Many therapists in the focus group suggested that a videoconferencing function could address the absence of visual cues of text-based online support groups.

## Discussion

AICF is a novel textual analysis system that tracks emotions in the texts expressed by online support group participants. To date, there is no similar AI system of this kind in the literature. Our study objectives were to investigate whether AICF added value to virtual care and to inform best virtual clinical practices by using real-time analytics, leading to greater ease and effectiveness for virtual support group management. When AICF functions are complemented by the basic functions of the chatroom platform, such as emoji check-ins and engagement alerts, our therapists found that AICF provided a new level of detail in tracking patient emotions and their engagement levels. Surprisingly, therapists prefer the emoji check-in, a non-AI item originally designed for researchers to provide a point of reference for real-time patient emotional experiences, over the distress warning. They felt that the emoji check-in was incredibly useful and undistracting for the patients. The next step is designing the display of participant emojis for maximum efficiency and aesthetics to provide actionable insights for therapists.

The original idea and purpose of developing an AICF dashboard was to give therapists essential indicators when facilitating a text-based online support group. This aim may be particularly helpful when a group leader is acting as the sole facilitator and when it is not possible to track patients’ bodily or facial cues. However, therapists suggest that there is a need to balance what type and amount of information is provided during group sessions. For example, therapists may find too much information (eg, for each group participant) overwhelming while conducting a group session. Distress warnings are viewed as helpful but can also be distracting, and for some, they may pose additional burden concerning legality issues. The literature suggests that health care providers may prefer positive feedback from an AI system instead of being warned about their potential mistakes [30]. The perception that there may be information that could be used legally may pose a barrier for mental health care providers in adopting AI technology in their clinical practice [31]. Our study found that therapists would like to maintain a high level of control over the AI functions, for example, discounting scores from the participants who dropped out of the session early and the content of the automated resource recommendations. Therefore, the AICF dashboard may require further refinement in order to provide ease of use and adaptability into practice. Recommendations included a dashboard that does not pose too much added burden or stress, is easy-to-understand, and that leads to or helps provide actionable insights. Specific suggestions include the placement of the essential graphics, developing a threshold to show extreme distress that signals clinical actions, and easy control over the automated functions.

A previous study [4] reported an AI system that automatically processes the transcripts of therapy sessions to generate a fidelity score for motivational interviewing. A focus group was conducted in that study with cognitive behavioral therapists regarding the system’s acceptability, appropriateness, and feasibility after watching a demonstration video of the technology. The feedback was generally positive. Similar to the findings in our study, therapists questioned the ability of detecting nonverbal cues and group cohesion. Similar to the concerns regarding our distress warning, the therapists in that study were also concerned about receiving low scores and how this would affect their self-perceived competence. With respect to ethical liability, the therapists also wanted to have more transparency on how the fidelity scores were calculated based on the session content.

The recent public health restrictions due to the COVID-19 pandemic served as an impetus for digital transformation in addressing mental health needs virtually. Consequently, digital means have become the main mode of mental health service delivery [32]. Moreover, privacy and confidentiality concerns over web-based teleconference calls have greatly lessened for most patients. Although CancerChatCanada group offerings and attendance [33] suggest positive experiences and good uptake with text-based groups, the therapists in the focus groups suggested that their group patients often expressed preference for teleconferencing, for example, cloud-based videoconferencing meeting over a text-based platform. Future research could consider assessing how to process transcripts generated by videoconferencing software for real-time analytics. Research efforts should also include the analysis of videos to track emotional states and the level of engagement of online group participants. Although AICF can be further refined, our findings have implications on exploring real-time voice-to-text technology and facial expression emotion analysis technology in a videoconferencing software.

An interesting point raised by the therapists is that AICF should be able to detect healthy and unhealthy emotions. The pressure to feel a need to only express positive emotions can occur in a group, including text-based groups, and may inhibit the expression of negative emotions, including sadness. This pattern can occur in both text-based and in-person groups and influence group participants to feel the need to remain positive to mask their negative feelings [29,34]. This response can result in feelings of isolation and further unmet needs and prevent open and authentic expression of emotions [35]. Therefore, further research is required to improve training of the algorithm to identify individuals who display unusual levels of expression of positive emotion in the context of cancer support groups.

Although AICF only showed a weak correlation with the patient-selected emoji scales, LIWC did not show any significant relationships. The lack of significant or consistent correlations among AICF, LIWC, and self-report IES-R is similar to that reported in other studies. A recent study found that LIWC emotion scores were not significantly associated with self-reports of emotional experience in the general population [36]. In another study addressing patients with subclinical depression, no correlation was found between the self-reported survey and the LIWC negative emotion score [37]. Lastly, in a study where patients were asked to watch a sad video, their self-reported emotions and LIWC scores were not significantly associated [38]. These findings suggest that patients do not express their emotional state verbally, indicating that analyzing textual data for emotions may be insufficient. The findings also imply that a static measure of emotions is not a good representation of a patient’s real-time emotional state. The Internet of Things appears promising for capturing relevant emotional and clinical outcomes of patients in real time. Wearable watches or sensors are gaining popularity to measure biometric and clinical outcomes such as heart rate variability, blood pressure, heart rate, skin temperature, galvanic skin responses, and goosebumps [39,40]. By leveraging the machine-learning signal processing algorithms and cloud-based computing services, we will be able to develop a novel way of detecting and tracking patient emotions and predicting clinical progress beyond analyzing textual data. Tracking emotions is an ethically complex subject; therefore, this type of study should strictly follow the informed consent process and comply with the protection of privacy and intimacy principle of data acquisition [41].

The functions of AICF, such as the text-based conversation summary, recommender system, engagement score, and group cohesion score, were helpful for tracking patient progress only if the information displayed in the dashboard was simple, undistracting, and free of possible legal liability. The basic emoji check-in seems to be the best way to track and show real-time reactions of the online group participants. Emotional analysis using facial cues during videoconferencing seems to be promising. Future studies will investigate the Internet of Things for clinical outcome evaluation and video analysis for emotion tracking.

## Appendix

Multimedia Appendix 1 Artificial intelligence–based co-facilitator methodology.

Multimedia Appendix 2 Artificial intelligence–based co-facilitator therapist interview guide.

## Abbreviations

AI: artificial intelligence
AICF: artificial intelligence–based co-facilitator
IES-R: Impact of Event Scale-Revised
LIWC: Linguistic Inquiry and Word Count
