# Supplementary material for: Therapist Feedback and Implications on Adoption of an Artificial Intelligence–Based Co-Facilitator for Online Cancer Support Groups: Mixed Methods Single-Arm Usability Study
Source: JMIR Cancer. 2023 Jun 9;9:e40113. doi: 10.2196/40113 (PMC10334721; doi:10.2196/40113)
Supplement: Multimedia Appendix 1 [file cancer_v9i1e40113_app1.docx]

### **Methods**

#### **Overview**

This project has 4 phases: (1) developing an AICF using a subset of existing CCC data based on PRIME, (2) evaluating its performance using human scoring, (3) beta testing the AICF within CCC, and (4) evaluating user experiences. This study will be conducted in compliance with the principles of the Declaration of Helsinki.

#### **Phase 1: Developing an AICF**

##### **Summary**

In addition to the 8 basic emotions detected by PRIME, the AICF will include additional functionality to support (1) real-time monitoring and alerting of emotional distress, participant engagement, and group cohesion, (2) participant emotional profiling, and (3) tailored resource recommendations.

‎**Figure S1. Functionalities of AICF. PRIME: Patient-Reported Information Multidimensional Exploration.**
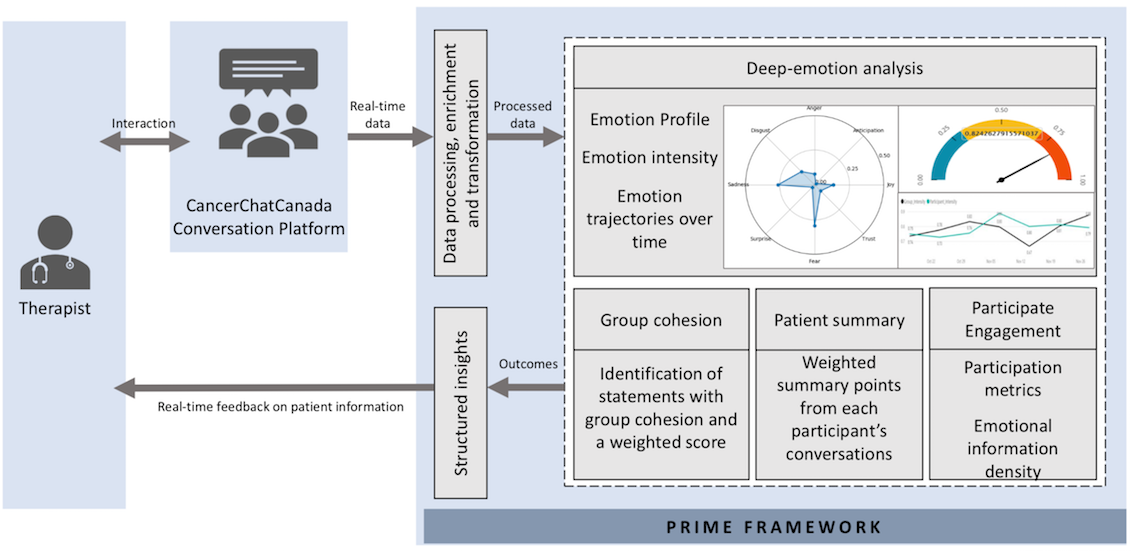


To inform development of the AICF, CCC data from 430 unique participants (approximately 80,000 conversations) across multiple sessions in 2 years will be used. The majority of participants were females, aged between 45 and 64 years, living in suburban or rural areas of British Columbia or Ontario. Many had breast cancer and were in the posttreatment period. See participant characteristics in Table 1.

The text data were deidentified to ensure confidentiality, using a risk-based approach in 2 steps. The first step used an open-source clinical text deidentification library (philter-ucsf [[6]](https://www.zotero.org/google-docs/?o2JndW)) to replace identifiers such as name, address, sex, age or year mention, and health care organization (hospital) names with asterisks, preserving word length. The second step involved human review and de-identification of any identifiers that the library missed. The AICF was trained using the de-identified data.

**Table S1. CCC Participant Characteristics**

| **Characteristics** | **n** | **%** |
| --- | --- | --- |
| **Gender (Female)** | 383 | 89.0 |
| **Age Group** |  |  |
| 18-24 | 6 | 1.5 |
| 25-34 | 27 | 6.2 |
| 35-44 | 66 | 15.3 |
| 45-54 | 130 | 30.2 |
| 55-64 | 147 | 34.2 |
| 65+ | 54 | 12.6 |
| **Location** |  |  |
| BC | 146 | 34.0 |
| Ontario | 142 | 33.0 |
| Alberta | 52 | 12.0 |
| Other provinces | 90 | 21.0 |
| **Geography** |  |  |
| Urban | 168 | 39.0 |
| Sub-urban/Rural Area | 262 | 61.0 |
| **Type of cancer** |  |  |
| Breast | 133 | 31.0 |
| Gynecological | 90 | 21.0 |
| Colorectal | 43 | 10.0 |
| Head and Neck | 13 | 3.0 |
| Other cancers | 151 | 35.0 |
| **Treatment Status** |  |  |
| Active treatment | 116 | 27.0 |
| Post-treatment | 176 | 41.0 |
| Unknown | 138 | 32.0 |

##### **Functionality 1: Real-Time Monitoring and Alert**

The AICF is designed to track emotional distress, participant engagement, and group cohesion. First, conversational extracts and user behaviors (eg, emotional intensity, participant engagement) will be used to train an ensemble of machine learning algorithms [[7]](https://www.zotero.org/google-docs/?hof7ku) that predict the likelihood of significant emotional distress. This ensemble method generates a weighted score that offsets potential bias in the 2 approaches as well as takes both emotion and engagement into account. Manually annotated texts of instances of distress were used to train the AICF. For the purpose of assessing the intensity of distress, 10 sessions of chat data were annotated and classified according to levels of distress (none, low, medium, or high) in the participants' statements. Based on the focus group feedback, the therapists highlighted that, apart from the 8 basic emotions, it is important to specifically detect participants' comments indicating hopelessness, distress, and loneliness, which are more complex emotional experiences specific to a cancer population [[8,9]](https://www.zotero.org/google-docs/?wyHpdf). Identifying these emotions are key in cancer online support groups as they are linked to depression (hopelessness, loneliness) [9] and poor outcomes (distress) [[10]](https://www.zotero.org/google-docs/?5aIA5H). Therefore, emotion intensities for all 11 emotions have been aggregated into group- and participant-specific emotion intensities. The AICF will produce risk scores of significant distress and that will be displayed and updated at each 30-minute interval on a 90-minute session timeline. If a participant’s risk score has increased significantly compared to the previous intervals, then the therapist will be alerted. The alert threshold will be finalized in the user testing phase.

Participant engagement is defined by the information density of their text posts, which consists of the emotional content detected by the AICF, number of words per post, and frequency of posting. The AICF will update every 5 minutes to show if participants are participating at high, medium, or low levels or if they have not posted in some time. Real-time monitoring of participant engagement, relative to the group will also be displayed on the real-time dashboard.

Group cohesion is defined as a sense of belonging to and feeling supported by the group [[11]](https://www.zotero.org/google-docs/?ImbevP). This is indicated by referring to other participants as “us,” “we,” and “our group”; expressing gratitude for sharing or help from others; commenting that they are looking forward to the next group; or chatting together outside of group time. The AICF will capture the group cohesive statements and present an aggregated score that denotes the level of group cohesiveness among all members. Group cohesive statements (100 examples) were annotated by the therapists from the CCC data, and keywords and phrases derived from these examples were used as seed terms in a word embedding model.

##### **Functionality 2: Participant Emotional Profiling**

The AICF will use features from PRIME to generate a collection of key phrases and topics and an emotional profile for each participant over a 90-minute session. The summary will consist of participant clinical and sociodemographic characteristics and will display key phrases that indicate emotional distress and the intensity [[12]](https://www.zotero.org/google-docs/?mLNzVb) of emotion associated with each concern on a 90-minute timeline. The display time window can be expanded as sessions continue.

##### **Functionality 3: Tailored Resource Recommendations**

The AICF will use outcomes from the first 2 functionalities to train an unsupervised incremental learning algorithm that merges similar participant profiles and differentiates dissimilar ones. The incremental learning feature will maintain these learned groupings (eg, pre surgery vs post surgery) over time, uncovering shared group behaviors and patterns. These will then be combined with known participant characteristics (eg, location, age), use of psychosocial resources (eg, sexual health clinic), and timing for a specific issue (eg, sexual health) from CCC data as inputs to a recommender system. Using association rule mining algorithms, a list of resources with predicted scores will be generated; resources with the highest predicted scores will be recommended to a participant [[](https://www.zotero.org/google-docs/?QzuR8r)13[]](https://www.zotero.org/google-docs/?QzuR8r). Test data will be used to evaluate relevance and reliability of each recommendation. This functionality will increase tangible support and service quality without incurring increased workload on the therapists.

#### **Phase 2: Human Scoring**

To develop the AICF, the emotion labeling outputs will be scored by a team of psychology undergraduate and graduate students, including 2 doctoral-level clinical psychology therapists. The team will score 20% of the output as feedback to retrain the AICF. This updated version will be run on the remaining 80% of the data. Each AICF version will be saved before it is trained on new data. To evaluate the AICF, 2 online support group groups’ messages will be withheld for the final testing phase of the AICF.

The team will score the output using guides based on definitions from the literature and examples from well-established psychometric measures (Table 2). For example, sadness and fear were selected for the first round of human scoring due to their relevance as symptoms of anxiety and depression, and they were the emotions in the high distress post category most commonly identified by annotators. The output will be scored at the sentence level, such that the target output must be clearly present in the sentence, without additional textual context.

**Table S2. Target measures for human scoring**

| **Output** | **Category** | **Description** |
| --- | --- | --- |
| Sadness | Emotion | Expressing loss, grief, unhappiness, hopelessness; feeling low, down or “blue”; behaviours such as crying, withdrawing. Possible indicator of depression (persistent, high distress) [[](https://www.zotero.org/google-docs/?fRAPZY)14[]](https://www.zotero.org/google-docs/?fRAPZY) |
| Fear | Emotion | Feeling scared, panicked, alarmed, apprehensive; less intensely worried, anxious, “stressed”, irritable, tense; being unable to focus, relax. Possible indicator of anxiety (persistent, high distress) [[](https://www.zotero.org/google-docs/?BYzOzx)14[]](https://www.zotero.org/google-docs/?BYzOzx) |
| Group cohesion | Group process | A culmination of participation, engagement, expressed mutual support, gratitude for other members, looking forward to future chat sessions and referring to the group as “we” and “us” to indicate a sense of belonging [[](https://www.zotero.org/google-docs/?myWqiW)11[]](https://www.zotero.org/google-docs/?myWqiW). |
| Emotional Profiling | Post-group feature | A text summary of key phrases with high emotional content for each participant, scored for accuracy. |
| Tailored Resource Recommendation | Post-group feature | A list of relevant psychosocial resources will be generated for each participant based on the conversations during the session. |

Using the literature, the scorers will note the instances in which the AICF has (1) correctly identified each output instance (true positive); (2) incorrectly identified an output instance (false positive); (3) correctly identified the lack of an output (true negative); or (4) missed an output in a sentence (false negative). For example, from the sentence “Yesterday I had a melt down, just felt so sad and cried, it came out of nowhere,” the AICF correctly identified the comment's sentiment as sadness and so did the human scorer (true positive). From the sentence “I can still laugh at some pretty bad jokes,” the AICF incorrectly identified the comment's sentiment as sadness, whereas the human scorer rated the comment's sentiments as no sadness (false positive). To judge the emotional intensities generated by the AICF, the scorers defined 4 levels of distress: low, moderate, moderate-high, and high.

Upon completion of the scoring, AICF performance will be evaluated for sadness, fear, and group cohesion measures using recall, precision, and F1 score. Precision is defined as a measure of result relevancy while recall is defined as a measure of how many truly relevant results are captured. The F1 score, which is the weighted average of precision and recall, takes both false positives and false negatives into account. For F1 scores below 80%, scorer feedback will be used to improve the AICF until it achieves 80%. The scoring results will be used to generate the list of keywords of queried expressions in the word embedding model, while linguistic rules will be added to handle exceptions such as negations, idioms, irony, or expressed sarcasms that are unique to participants with cancer. This feedback loop will improve the performance of each functionality using domain expertise [[](https://www.zotero.org/google-docs/?Rtabqv)15[]](https://www.zotero.org/google-docs/?Rtabqv) to produce an acceptable evaluation F1 score.

#### **Phase 3: Beta Testing**

##### **Summary**

The AICF will be deployed and tested in the CCC platform background (out of the therapists’ view) in 3 groups. It will then be run for therapist use and feedback for beta testing on 10-12 groups to analyze the performance of the AICF system output, such as all emotions expressed (including distress), intensity, group cohesion, engagement, and emotional profiling features (see Table 2). We hypothesize that the AICF output will be highly correlated with standard clinical measures of psychological outcomes and have high sensitivity and predictive values for distress. These quantitative evaluations will provide evidence to support the AICF’s validity and reliability.

##### **Design**

A single-arm trial to evaluate the AICF’s validity and reliability among CCC therapists and participants.

##### **Participants**

Ten therapists and 100 support group participants (ie, patients and caregivers) (10-12 groups) will be recruited through a multipronged approach, including in-person (University Health Network clinics), print (flyers and posters posted at University Health Network locations), and digital media (eg, Twitter, Google, and Facebook [[16]](https://www.zotero.org/google-docs/?RaPgkd), CCC platform, and webpages of CCC provincial partners across Canada). In-person recruitment will take place at University Health Network clinics using protocols approved by the University Health Network research ethics board. A study coordinator will explain the study prior to informed consent. For online and print recruitment, respondents will be provided with the study webpage and phone number of the research team for study inquiry. Interested patients will be followed-up by a call from the study coordinator for study details. Study log will be maintained and reasons for nonparticipation collected. The existing CCC therapist roster will be used to recruit therapist-participants directly. Therapists will receive training on the AICF. An estimated 120,000 posts (200 posts/user/session × 6 sessions × 100 users) will be generated, sufficient for the sensitivity and specificity analysis [[](https://www.zotero.org/google-docs/?RnV57U)17[]](https://www.zotero.org/google-docs/?RnV57U). This study has been approved by the University Health Network research ethics board.

##### **Measures**

Participant distress will be assessed by standardized measures pre and post program by several scales. The Impact of Event Scale-Revised [[26]](https://www.zotero.org/google-docs/?6G3gyG) is composed of 22 items rated on a 5-point Likert scale, yielding a total score ranging from 0 to 88—8 items on intrusion (Cronbach α=.87-.94), 8 items on avoidance (Cronbach α=.84-.87), and 6 items on hyperarousal (Cronbach α=.79-.91). The 7-item Hospital Anxiety and Depression Scale (HADS) [[18]](https://www.zotero.org/google-docs/?Ehxf3E), with items rated on a scale of 0 to 3, yielding a total score ranging from 0 to 21 has 2 subscales: anxiety and depression (Cronbach α=.88 and .92, respectively) [[18]](https://www.zotero.org/google-docs/?dMULtf). The 18-item Brief Symptom Inventory [[](https://www.zotero.org/google-docs/?DkQUBv)19[]](https://www.zotero.org/google-docs/?DkQUBv) is rated on a 5-point Likert scale, and the sum is transformed into a T-score. The Brief Symptom Inventory is composed of 3 dimensions: somatization, depression, and anxiety (Cronbach α=.71-.85) [[19]](https://www.zotero.org/google-docs/?Cp4wBu). A participant will be defined as having significant distress if they score above the cut-offs for any of the scales: cancer-related distress (Impact of Event Scale-Revised score >24), symptoms of depression (HADS score>10), or anxiety (Brief Symptom Inventory T-score >60).

Participant postsession emotionality will be assessed using the Positive and Negative Affect Schedule [[](https://www.zotero.org/google-docs/?zatx9s)20], a 20-item self-report measure of positive and negative affect (Cronbach α=.89 and .85, respectively). Postprogram group cohesion will be measured using the 19-item Therapeutic Factor Inventory [[](https://www.zotero.org/google-docs/?eq3anK)21]—Instillation of Hope (4 items), Secure Emotional Expression (7 items), Awareness of Interpersonal Impact (5 items), and Social Learning (3 items). The items are rated on a scale from 1 to 7 and a mean score is based on a sum of the item ratings multiplied by factor score weightings (Cronbach α=.71-.91)[[21]](https://www.zotero.org/google-docs/?O39fHn). Therapists will assess the AICF’s usability postsession using the 10-item System Usability Scale using a 5-point Likert scale of agreement with scores ranging from 0 to 100 (Cronbach α=.95)[[](https://www.zotero.org/google-docs/?QPJhAL)22]. Finally, the online support group experience will be measured using the 24-item Counsellor Activity Self-Efficacy Scale [[](https://www.zotero.org/google-docs/?fULZKX)23], with items rated on a 10-point Likert scale (Cronbach α=.96) [[23]](https://www.zotero.org/google-docs/?H5RL0R).

##### **Emoji Scale**

Emoji scales validated as representations of physical and emotional quality of life in cancer populations [[24]](https://www.zotero.org/google-docs/?jJTWFV) will be used to track emotions of the participants during each session. For AICF validation, we will employ an automatic check-in that will occur at three 30-minute intervals using 9 different emoticons (eg, worried, sad, supported) from which participants can choose to represent their emotional states in the moment. Each participant will provide up to 300 emoji ratings (3×10 sessions).

##### **Statistical Analysis**

The ability of the AICF to correctly identify distress will be assessed. First, a chi-square test will be used to assess the sensitivity and specificity of the AICF against the self-reported emoji at the 30-minute interval. Second, linguistics inquiry word count [[](https://www.zotero.org/google-docs/?yc4kXH)2[7]](https://www.zotero.org/google-docs/?yc4kXH) will be performed on each post. Linguistics inquiry word count scans each post for the linguistic markers of distress (eg, first-person singular pronouns, and words that Linguistics inquiry word count classifies as sad, anxiety or fillers) and provides a correlation coefficient. We hypothesize that correlations between linguistics inquiry word count and AICF output would be strong (≥0.7). Third, a precision-recall curve (positive predictive value vs sensitivity) will be used to map AICF classifications against established cut-offs of a standardized measure. An area under the curve >80% would be considered high performance [[](https://www.zotero.org/google-docs/?dmDCaQ)25[]](https://www.zotero.org/google-docs/?dmDCaQ). The precision-recall curve can be used to inform the statistical threshold for distress that warrants a therapist alert. Fourth, construct (convergent) validity of AICF sentiment analysis will be compared against self-reported standardized measures. We hypothesize that all positive and negative emotions extracted will be strongly correlated (Pearson correlation coefficient ≥0.7) with Positive and Negative Affect Schedule subscales scores. For example, the extracted negative emotions will be positively correlated with HADS. Finally, internal consistency will be measured among extracted negative and positive emotions using Cronbach α.

#### **Phase 4: Evaluation of User Experiences**

Participants will rate their satisfaction with their online support group experience (eg, group cohesion) using the Therapeutic Factor Inventory. Therapists will provide ratings of system usability (with the System Usability Scale) and perceived self-efficacy measure (with the Counsellor Activity Self-Efficacy Scale) in leading the groups. All of these ratings are expected to be high (>80th percentile). Quality indicators, such as study attendance and dropout rate (defined as participants missing more than 2/10 sessions) will be compared using chi-square tests to those in a special topic group (eg, sexual health group). We expect that the dropout rate will be 50% less than those of existing groups.
