# Supplementary material for: Therapist Feedback and Implications on Adoption of an Artificial Intelligence–Based Co-Facilitator for Online Cancer Support Groups: Mixed Methods Single-Arm Usability Study
Source: JMIR Cancer. 2023 Jun 9;9:e40113. doi: 10.2196/40113 (PMC10334721; doi:10.2196/40113)
Supplement: Multimedia Appendix 2 [file cancer_v9i1e40113_app2.pdf]

Building an Artificial Intelligence System to Enhance Online Support Groups in Cancer

Date: July 20th
Time: 12:00 pm EST
Location: Teams Meeting

Objective:

To gain feedback from CCC therapists (end-users of AICF) on AICF system

Agenda:

Time	Activity	Duration
12:00	Opening Remarks	5 min
12:05	Introductions<br>• Where do you practice?<br>• What's your specialty?<br>• What are the Cancerchat groups that you have facilitated?	10 min
12:15	Part 1: Review and Feedback on the Previous Focus Group Findings (10 min)	
	Display the previous findings	2 min
	Does it resonate with you?	8 min
12:25	Part 2: Presentation: AICF design - Benefits and Challenges (10min)	
	Presentation	5 min
	Group discussion	5 min
12:35	Part 3: Opinion on the AI system (25 min)	
	Display and explain AI system - Session summary, In session distress warning, Resource recommender system	3 min x 4
	Group discussion in each area	5 min x 4
	Wrap up and summary	

Questions:

PART 1: Review and Feedback on the Previous Focus Group Findings (10 mins)

Question	Prompts
• Review the previous focus group findings; anything to add?	• Have you changed the way you managed the support groups?
• What is the most difficult aspect of managing an online support group?<br>a. Do you feel AICF makes managing an OSG easier?<br>b. What aspects does AICF make easier?	For example:<br>• Does it help with facilitation?<br>• Distress, engagement, warnings, concerns?<br>• Are there any issues?<br>• Are there any distractions?

PART 2: Benefits and Challenges or Limitations of AICF (20-30 min)

• In what aspects do you find the AICF useful?	• Distress warnings<br>• Low engagement warnings<br>• Recommender system for psychosocial concerns<br>• Dashboard
• In what aspects do you find the AICF limited?	• Emoji check-ins (research purpose)<br>• Dashboard<br>• Engagement warnings

Part 3: Recommendations to the specific functionalities of AICF (20-30 min)

<table>
<tr><td>Question</td><td>Prompts</td></tr>
<tr><td>1. Dashboard</td><td></td></tr>
<tr><td>Now that you’ve seen the dashboard, do you find it useful? Relevant? Self explanatory? Distracting? Overwhelming?
During the session?
Before and After session?
What do you like/not like about the after session report? What is most useful to you?</td><td>Radar graphs
Emotional change over time
Group cohesion outcome
Distress over sessions line graph
Compare someone session over session
Compare someone to the group average
Tracking specific emotions with its intensity during a session
Tracking multiple emotions during a session
Emotions that are extreme (e.g., severe distress)
Indicating when someone is “silent” or withdrawn from the group</td></tr>
<tr><td colspan="2">2. DISTRESS Warnings</td></tr>
<tr><td>In what aspects do you find the distress warnings useful?
Do you feel like AICF picks up on distress more/less than actually present?</td><td>During the session?
Missed distress?</td></tr>
<tr><td>3. RECOMMENDER SYSTEM</td><td></td></tr>
<tr><td>Do any participants discuss the usefulness of the recommended resources?
Do these resources accurately represent their concerns?</td><td>Relevant?
Discussion based on resources?</td></tr>
</table>

• Do you feel the resource recommender is a helpful tool for you as a facilitator?	

PART 4: Overall impression of AICF System (10-20 min)

Question	Prompts
• Is there anything you would like to see added or removed from the AICF system?<br><br>a. Suggestions on additional human-machine interactions to improve the overall effectiveness of online groups	For example:<br>• In the dashboard<br>• After session reports
